# Biofluorescence in Catsharks (Scyliorhinidae): Fundamental Description and Relevance for Elasmobranch Visual Ecology

**DOI:** 10.1038/srep24751

**Published:** 2016-04-25

**Authors:** David F. Gruber, Ellis R. Loew, Dimitri D. Deheyn, Derya Akkaynak, Jean P. Gaffney, W. Leo Smith, Matthew P. Davis, Jennifer H. Stern, Vincent A. Pieribone, John S. Sparks

**Affiliations:** 1 Baruch College, City University of New York, Department of Natural Sciences, New York, NY 10010, USA; 2 City University of New York, The Graduate Center, Program in Biology, New York, NY 10016, USA; 3 American Museum of Natural History, Sackler Institute for Comparative Genomics, New York, NY 10024, USA; 4 College of Veterinary Medicine, Cornell University, Ithaca, NY 14853, USA; 5 University of California, San Diego, Scripps Institution of Oceanography, La Jolla, CA 92093, USA; 6 University of Haifa, Charney School of Marine Sciences, Haifa, 3498838, Israel; 7 Interuniversity Institute of Marine Sciences, Eilat, 88103, Israel; 8 University of Kansas, Biodiversity Institute and Department of Ecology and Evolutionary Biology, Lawrence, KS 66049, USA; 9 St. Cloud State University, Department of Biological Sciences, St. Cloud, MN 56301, USA; 10 The John B. Pierce Laboratory, Cellular and Molecular Physiology, Yale University School of Medicine, New Haven, CT 06519, USA; 11 American Museum of Natural History, Division of Vertebrate Zoology, Department of Ichthyology, New York, NY 10024, USA

## Abstract

Biofluorescence has recently been found to be widespread in marine fishes, including sharks. Catsharks, such as the Swell Shark (*Cephaloscyllium ventriosum*) from the eastern Pacific and the Chain Catshark (*Scyliorhinus retifer)* from the western Atlantic, are known to exhibit bright green fluorescence. We examined the spectral sensitivity and visual characteristics of these reclusive sharks, while also considering the fluorescent properties of their skin. Spectral absorbance of the photoreceptor cells in these sharks revealed the presence of a single visual pigment in each species. *Cephaloscyllium ventriosum* exhibited a maximum absorbance of 484 ± 3 nm and an absorbance range at half maximum (λ_1/2max_) of 440–540 nm, whereas for *S. retifer* maximum absorbance was 488 ± 3 nm with the same absorbance range. Using the photoreceptor properties derived here, a “shark eye” camera was designed and developed that yielded contrast information on areas where fluorescence is anatomically distributed on the shark, as seen from other sharks’ eyes of these two species. Phylogenetic investigations indicate that biofluorescence has evolved at least three times in cartilaginous fishes. The repeated evolution of biofluorescence in elasmobranchs, coupled with a visual adaptation to detect it; and evidence that biofluorescence creates greater luminosity contrast with the surrounding background, highlights the potential importance of biofluorescence in elasmobranch behavior and biology.

Although biofluorescence has been extensively examined in cnidarians, particularly corals^1^,^2^,^3^, the phenomenon has only recently been shown to be phylogenetically widespread and phenotypically variable in both cartilaginous and bony marine fishes^4^. Biofluorescence has even recently been observed in sea turtles^5^. This revelation leads to many new questions regarding the biological and ecological role of biofluorescence in the marine realm, because fishes and sea turtles, unlike cnidarians, possess advanced visual systems^6^,^7^,^8^,^9^.

Marine organisms biofluoresce by absorbing the dominant higher energy ambient blue light via fluorescent compounds, and reemit it at longer, lower energy wavelengths, visually resulting in green, orange, and red fluorescence to the human visual system. With increasing depth in the ocean, the spectral quality of sunlight becomes restricted to a narrow range of wavelengths of blue light and intensity decreases in an approximately exponential manner^10^. In clear ocean water the light spectrum bandwidth progressively narrows with increasing depth, reaching a wavelength peak of 465 nm and a narrow bandwidth of ~20 nm at the maximum depth of penetration^11^. Thus, biofluorescence adds photons in a spectral region (i.e., longer wavelengths of the visible spectrum) not otherwise represented at depth, which has been shown to drive evolutionary shifts in light absorbance across many taxa, such as the evolution of divinyl-chlorophyll a in marine micro algae^12^.

Green Fluorescent Protein (GFP) was discovered in a hydrozoan jellyfish, *Aequorea victoria*, coupled to the bioluminescent apparatus, where it converts blue bioluminescent light to green light^13^. The GFP family has since proven to be one of the most useful tools in biomedical science^14^. GFP orthologs have also been reported in non-bioluminescent anthozoans^2^,^15^,^16^, planktonic copepods^17^, and lancelets^18^. In fishes, a novel bilirubin-binding fluorescent protein (FP) was found in *Anguilla japonica*^19^,^20^, and it has been shown that bilirubin-binding FPs also occur in cryptic marine eels of the genus *Kaupichthys*^21^. This new family of eel FPs evolved as the sister group to brain fatty acid-binding proteins^21^.

Some fishes exhibit strong interspecific variation in fluorescent emission patterns (e.g., members of the lizardfish genus *Synodus* and the goby genus *Eviota*), which has led to the hypothesis that biofluorescence functions as a form of species recognition^4^, akin to the unique bioluminescent signals produced by both shallow water and deep-sea fish lineages^22^,^23^,^24^. There are also many fishes known to possess yellow intraocular (lenses or cornea) filters^25^, which potentially function as long-pass filters and enable enhanced perception of fluorescence. Biofluorescence has also been shown to play a role in the behavior of marine organisms: fluorescence at the tips of the tentacles in the hydromedusa *Olindias formosa* attracts juvenile rockfishes of the genus *Sebastes*^26^; the fairy wrasse, *Cirrhilabrus solorensis,* responds to red biofluorescence^27^; and fluorescence in the mantis shrimp, *Lysiosquillina glabriuscula*, enhances signaling 28.

Although recent work has demonstrated that biofluorescence is widespread in marine cartilaginous and ray-finned fishes^4^,^21^, very few studies have examined the fluorescent properties of these lineages. Further assessment of these spectral properties is critical to advancing our understanding of the evolution of biofluorescence in marine vertebrates and its potential impact on their evolution, behavior, diversification rate, and the composition of marine ecosystems in general. Here we focus on cartilaginous fishes and examine the photoreceptor cells in two species of catsharks (Scyliorhinidae: *Cephaloscyllium ventriosum* and *Scyliorhinus retifer*) that exhibit bright green fluorescence patterns resulting from the presence of fluorescent compounds in their skin. We performed hyperspectral imaging of their skin and investigated how the green-dominated fluorescence affected the appearance of these sharks to conspecifics given their spectral sensitivity and the blue ambient water background. We also assembled a “shark-eye” camera and imaged catsharks in their natural environment with filters closely matching the absorbance spectrum of the measured visual pigment, as well as with underwater fluorescence photography. Models for estimating the lightness perception of these monochromatic sharks do not exist, therefore, we used models developed for the human visual system to gain insight into how biofluorescence changes the appearance of the sharks in the eyes of their conspecifics, compared to the way they appear under white light.

## Results

**Figure 1:**
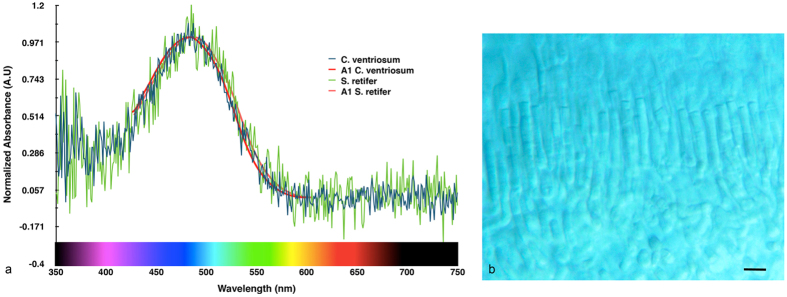
(**a**) Microspectrophotometry data from *Celphaloscyllium ventriosum and Scyliorhinus retifer.* Maximum absorbance of 484 and 488 nm, respectively. (**b**) Long rods of *S. retifer* (scale =10 μm).

**Figure 2:**
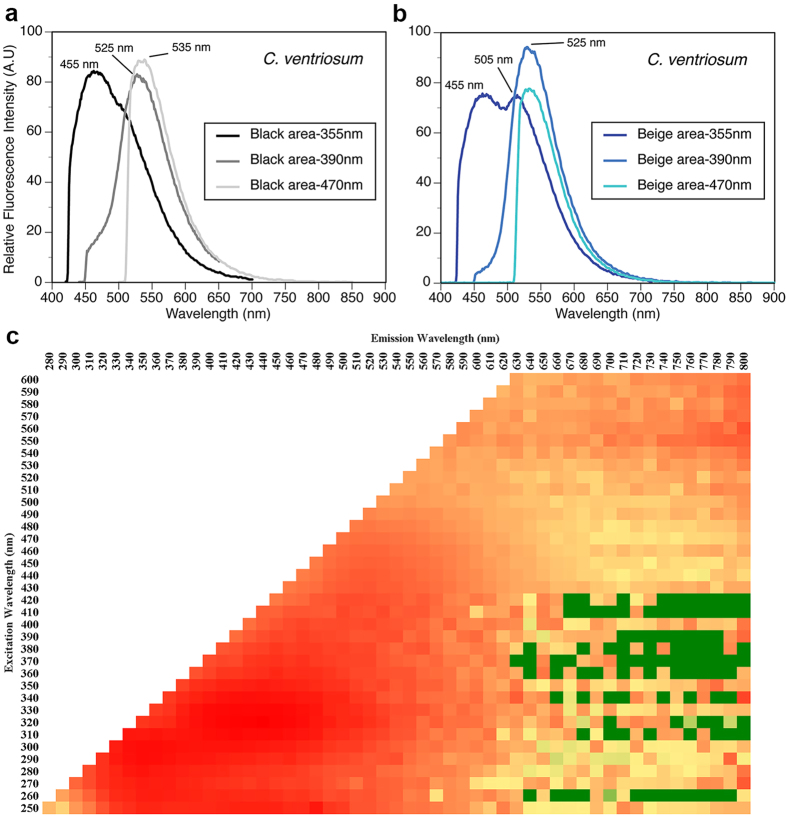
Spectral characterization of *C. ventriosum.* (**a**) Fluorescence spectra following excitation at 355, 390, and 470 nm from black areas of the skin, using hyperspectral imaging analysis. (**b**) Same analysis from beige areas of the skin. (**C**) Excitation emission matrix (log scale representation) after extraction of fluorescent compounds from skin in methanol solvent, which included both black and beige areas.

**Figure 3:**
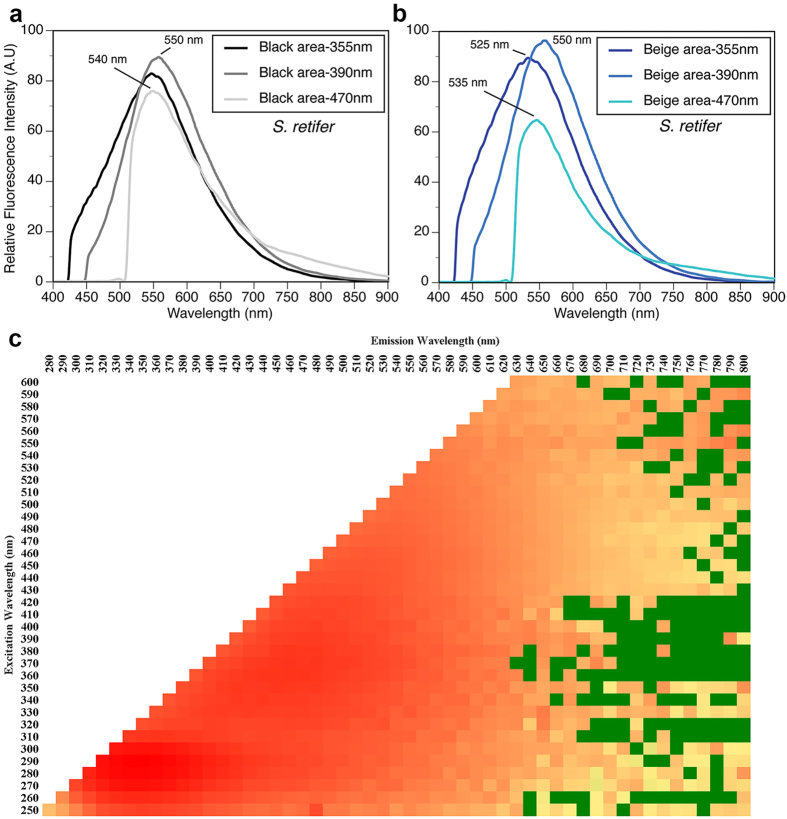
Spectral characterization of *S. retifer.* (**a**) Fluorescence spectra following excitation at 355, 390, and 470 nm from black colored areas of the skin, using hyperspectral imaging analysis. (**b**) Same analysis from beige colored areas of the shark skin. (**c**) Excitation emission matrix (log scale representation) after extraction of fluorescent compounds from skin in methanol solvent, which included both black and beige areas.

**Figure 4:**
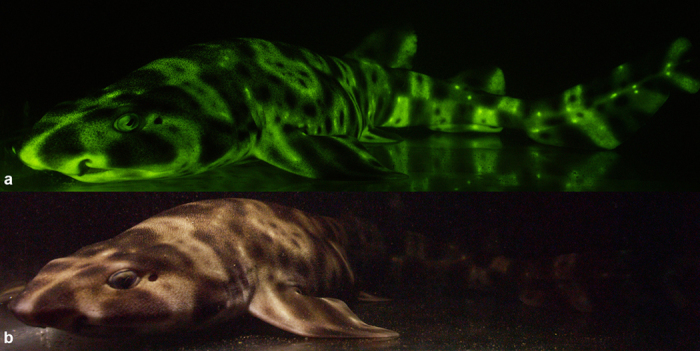
(**a**) Fluorescent (excitation 450–500 nm; emission 514 LP), and (**b**) white light image of a 54.0 cm female swell shark (*Cephaloscyllium ventriosum*).

**Figure 5:**
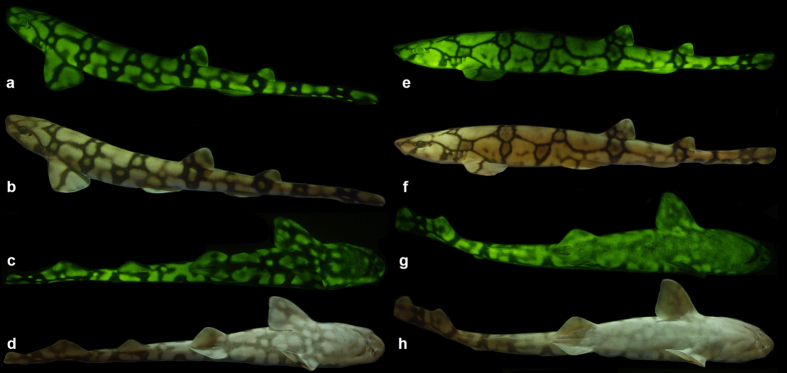
(**a–d**) Fluorescent and white light pigmentation pattern of a female chain catshark (*Scyliorhinus retifer*, 32.2 cm; and (**e–h**) of a male *S. retifer* (26.4 cm). Males have pelvic claspers that fluoresce, whereas the females lack claspers, and the reticulated pigmentation pattern is more pronounced in females (thicker dark black/brown lines), particularly ventrally, under both fluorescent and white lighting.

Microspectrophotometry (MSP) conducted on the retina of *Cephaloscyllium ventriosum* revealed a single visual pigment with an absorbance maximum (λ_max_) at 484 ± 3 nm and an absorbance range at half maximum (λ_1/2max_) of 440–540 nm. *Scyliorhinus retifer* was also found to possess a single visual pigment with a λ_max_ of 488 ± 3 nm and a λ_1/2max_ of 440–540 nm (Fig. 1). *C. ventriosum* and *S. retifer* were imaged and epidermal tissue from the flattened dorsal side of their head spectrally analyzed using a hyperspectral imager in fluorescence mode (Figs 2 and 3). The dermal patches of *C. ventriosum* and *S. retifer* that were imaged always included one of the darker/black reticulated patches (in bright field) and light beige areas, which is the typical repeating, reticulated pigmentation pattern characteristic of these catsharks (Figs 4 and 5). Fluorescence images clearly showed that at 355 nm excitation, bright blue fluorescence (455 nm emission [em.] with a shoulder towards 500 nm; Figs 2 and 3a) was associated with the darker areas of the skin, whereas some dim blue-green fluorescence (455–505 nm em.; Figs 2 and 3b) was observed in the lighter beige regions of the skin. When excited at 390 and 470 nm, the fluorescence images show that only intense green fluorescence (525–535 nm em.) was generated from both the darker and lighter pigmented areas of the skin. Green fluorescence was most intense from the lighter beige colored areas (Figs 4 and 5). When analyzed via hyperspectral mapping, the blue and green emission spectra from the shark skin were clearly spatially separated and mapped to the darker areas (blue emission spectrum) and beige areas (green emission spectrum), respectively.

**Figure 6:**
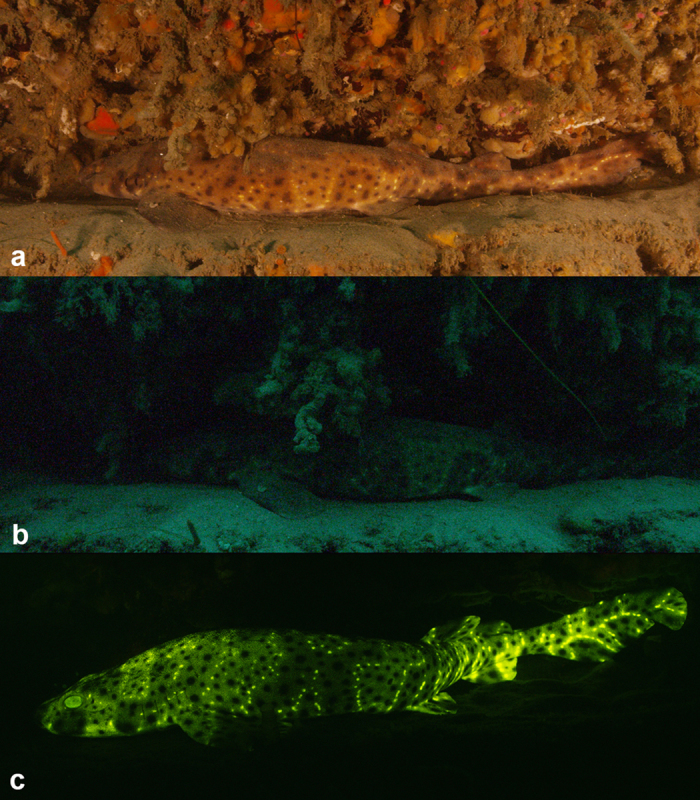
*Cephaloscyllium ventriosum* in its natural environment in Scripps Canyon (San Diego) under (**a**) white light; (**b**) natural light; (**c**) when excited with 450/70 nm calumniated lighting and imaged with a 514 nm long-pass emission filter.

These analyses conducted directly on the skin were complemented by spectral analysis of skin extracts after immersion in different solvents (methanol, acetone, dichloromethane), which also showed fluorescence in the blue and green spectral range when excited at 355 nm and 390 nm, respectively. These results confirmed the occurrence of two biofluorescent compounds (or the same compound in two different chemical forms) in distinct areas of the skin corresponding to the darkly pigmented areas versus light beige regions when observed in bright field. The presence of patches of fluorescence suggests that the observed fluorescence does not result from a common cellular constituent (i.e. keratin). *Cephaloscyllium ventriosum* possesses bright beige spots (Figs 4b and 6a), which correspond to significantly higher (>100–150×) levels of green fluorescence intensity (data not shown), than the darker pigmented, reticulated portions of the body (Figs 4a and 6c). Whether these spots result from increased concentration of the particular green fluorescent protein/compound or from a decrease of skin pigment locally remains to be determined.

The substance responsible for fluorescence was extracted in methanol (shown to be the best of the various solvents tested) before being analyzed for spectral excitation and emission characteristics, and depicted in an Excitation Emission Matrix (EEM). Skin tissues for extraction were obtained from the dorsal side of the head, where the skin can be more evenly and easily removed because of the hardness of the underlying skull. Darker patches did not show a clearly defined line separating them from the lighter beige patches, but instead showed a gradual increase in darkness moving toward the patch. In this gradual transition between beige and darker patches, fluorescence was still observed from under the dark pigments, as confirmed from the observation of fluorescence in cross-section (Figs S2 and S3). This suggests that the fluorescence is clearly muted by the pigments of the darker patches, whereas the same level of the fluorescent substance can be found throughout the epidermis (Figs S2 and S3). In *C. ventriosum* the fluorescent substance exhibited a wide range of excitation, ranging from 250–450 nm (yellow area in EEM; Fig. 2c) and peaking between 280–350 nm (dark orange/red area in EEM; Fig. 2c). Likewise, it had a broad emission peak ranging from 300–600 nm and peaking between 320–490 nm (Fig. 2c). Overall, the excitation spectrum was similar in *S. retifer,* ranging from 250–550 nm (yellow area in EEM; Fig. 3c) and peaking between 270–300 nm (dark orange/red area in EEM; Fig. 3c). Fluorescent emission in *S. retifer* ranged from 300–600 nm, peaking between 310–390 nm (Fig. 3c).

### *In-situ* Imaging

**Figure 7:**
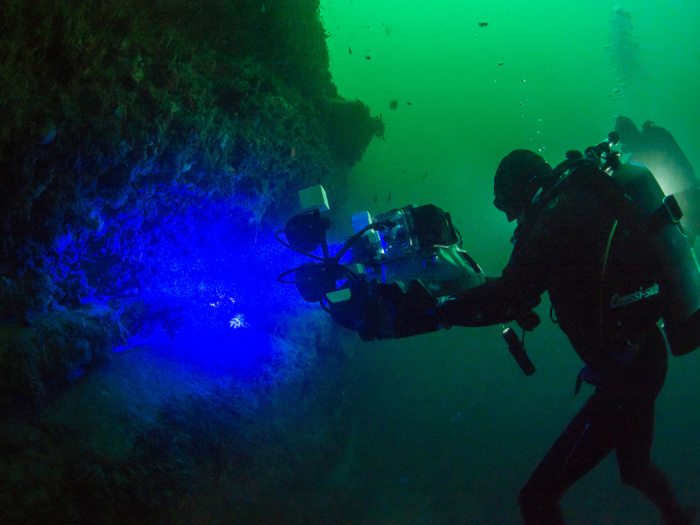
Scientific biofluorescent imaging camera and lighting system developed to obtain 4 K imagery shown underwater in Scripps Canyon, San Diego, CA. Image courtesy of Kyle McBurnie.

**Figure 8:**
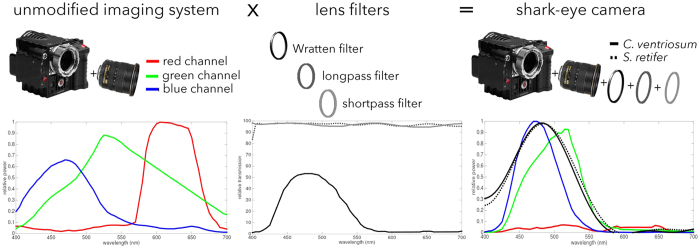
“Shark-eye” imaging was done by fitting three lens filters on the Red Epic camera, and keeping the blue channel response of the resulting system. Camera sensor response after the placement of the lens filters closely matches the visual pigment of *Cephaloscyllium ventriosum* and *Scyliorhinus retifer*. The transmission through the lens was assumed to be 100% for practical purposes.

**Figure 9:**
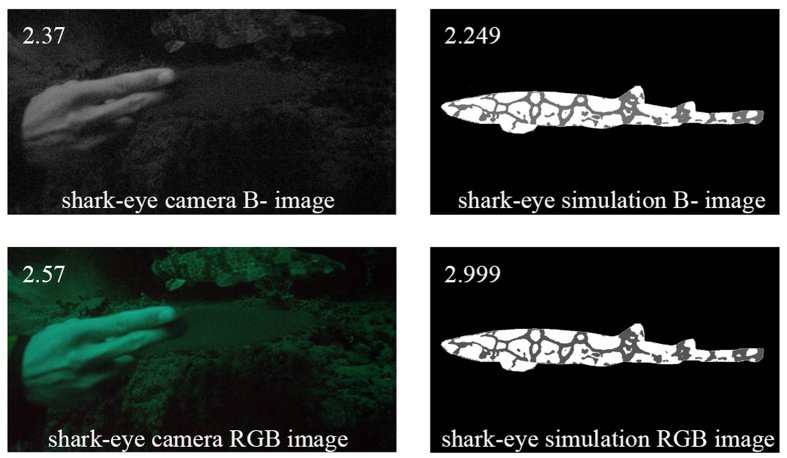
Beige/black patch intensity between the actual “shark-eye” camera, and our simulation. Image taken while holding an underwater spectra paper.

**Figure 10:**
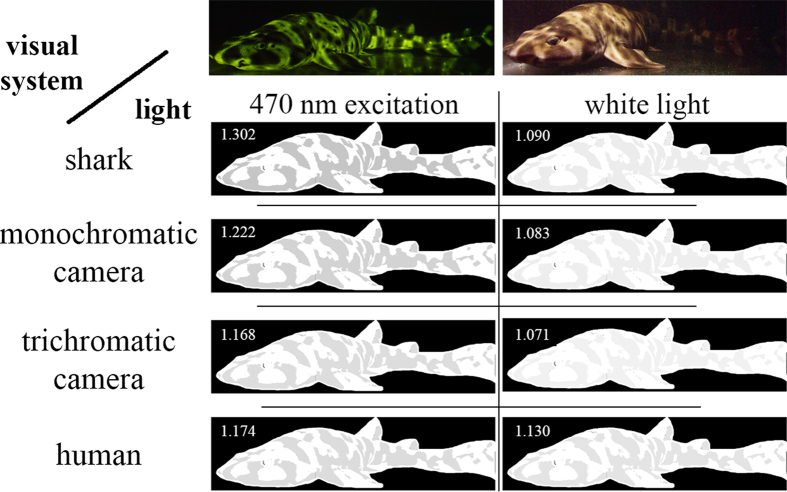
*Cephaloscyllium ventriosum* model of the shark eye as a monochromatic human with the 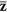 spectral sensitivity curves given in Fig. 1. Reflectance spectra for darkly pigmented and beige skin components of each shark species from Fig. 2 taken under white light, and emitted after exposure to 470 nm monochromatic light. Radiance and fluorescence spectra for dark and beige skin components of each shark species were converted into XYZ tri-stimulus values and then to sRGB color space^30^.

**Figure 11:**
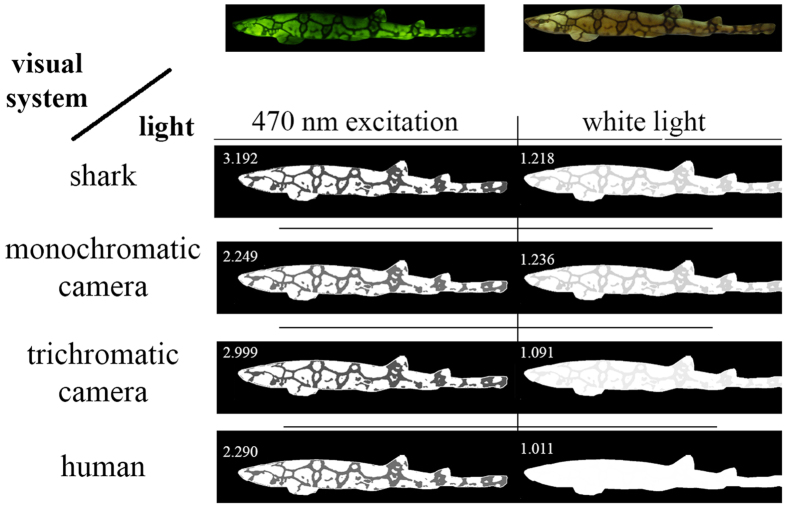
*Scyliorhinus retifer* model of the shark eye as a monochromatic human with the 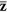 spectral sensitivity curves given in Fig. 1. Reflectance spectra for dark and beige skin components of each shark species from Fig. 2 taken under white light, and emitted after exposure to 470 nm monochromatic light. Radiance and fluorescence spectra for dark and beige skin components of each shark species were converted into XYZ tri-stimulus values and then to sRGB color space^30^.

We imaged *C. ventriosum* in their natural environment using only ambient lighting; with a “shark-eye” camera that closely matched the absorbance spectrum of the visual pigments of *C. ventriosum and S. retifer*; and with a lighting and camera system equipped to excite fluorescence and only capture the emitted fluorescent spectral range produced by the animal (Fig. 7). Under normal ambient underwater lighting conditions and without the use of lights or filters, *C. ventriosum* was almost not visible against the canyon wall (Fig. 6b, Supp. Fig. 1). When imaged with a filter pack closely matching the absorbance spectrum of the visual pigment of *C. ventriosum* using only natural light (Fig. 8), luminosity contrast was created on areas where the most intense fluorescence is distributed on the shark (lighter skin areas) compared to the rest of the body (Fig. 9). This was also supported by our RGB simulations of both *C. ventriosum* (Fig. 10) and *S. retifer* (Fig. 11).

### Aquarium and Laboratory Imaging

Cross-sectional images of the two catshark species show the fluorescent material to be localized solely on the dermis and the lens of the eye (Supp. Figs 2 and 3). Fluorescence was also observed in the egg cases of both species (Supp. Fig. 4). *Cephaloscyllium ventriosum* has small intensely green fluorescent spots over much of the body, which appears light beige under white light. Females also have a unique “face mask” with light spots in the center on each side and more dense ventral spotting that extends further anteriorly than in males. *Scyliorhinus retifer* exhibits an alternating light and dark reticulated pigmentation pattern (Fig. 5), but it lacks the brightly fluorescent spots characteristic of *C. ventriosum* (Fig. 4). In *S. retifer*, females, the reticulated pigmentation pattern is more pronounced (i.e., thicker dark brown/black lines), particularly ventrally, under both fluorescent and white lighting (Fig. 5).

### Modeling of visual pigments that best discriminate shark biofluorescence

**Figure 12:**
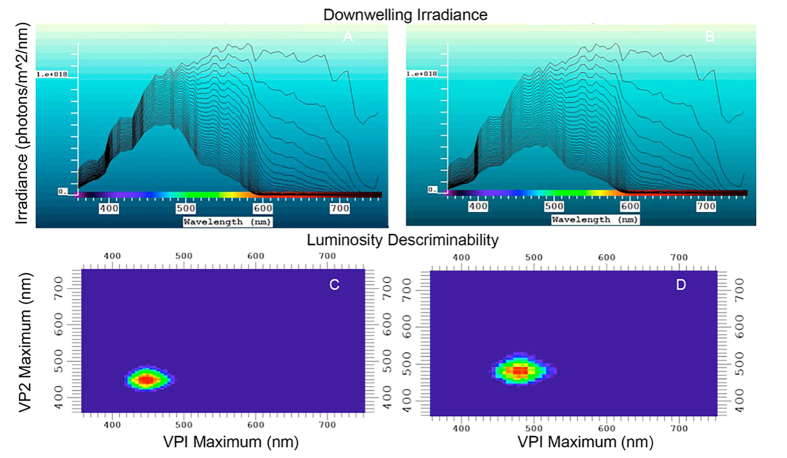
Calculated downwelling irradiance curves from 1 meter to 30 meters depth in (**A**) oligotrophic blue water, (**B**) more productive blue/green water that a visual pigment for 485 nm (such as in *C. ventriosum*) would have been best matched^29^. (**C**) Locus of the visual pigments best able to discriminate targets against the background based on luminosity at 30 meters depth in blue water, and (**D**) in blue/green water. In both cases, the locus is such that quantum catch in the given water type is matched as expected from the “contrast hypothesis.” Red loci are the optimal with those of other colors showing decreasing optimization.

The model developed by Loew and Zhang^29^ was employed to calculate which visual pigment(s) would best discriminate between green fluorescence and gray targets based on luminosity contrast using the background spacelight estimated at increasing depths within Scripps Canyon (San Diego County, California), where several *C. ventriosum* were imaged (at ~30 m depth; Fig. 12). As with all such models, an iterative procedure was used to reduce irradiant intensity while varying the λ_max_ of visual pigments, taking into account the properties of the water using a derived equation of radiative transfer. The model showed that even at the upper reaches of their habitat (e.g., 30 m where *C. ventriosum* was filmed *in situ*), it was possible for their visual apparatus to detect blue/green fluorescence.

An analysis was conducted to determine what water color was optimal for the 484 nm (and 488 nm of *S. retifer*) visual pigment, along with the best-tuned pigments for discriminating gray targets in clear, blue oceanic water (i.e., no chlorophyll or dissolved organic matter). The 484 nm and 488 nm pigments are better matched for bluer, deeper water. However, the visual pigment of *S. retifer* is still able to discriminate green biofluorescence in all oceanic or in-shore waters, as long as the target is close enough and there are sufficient photons to stimulate fluorescence above background noise in that part of the spectrum.

Another analysis was conducted where radiance and fluorescence spectra for dark and beige skin components of each shark species were converted into XYZ tri-stimulus values and then to sRGB color space^30^. Under these conditions we find that fluorescence creates greater luminosity contrast for both *C. ventriosum* (Fig. 10) and *S. retifer* (Fig. 11), a result also confirmed *in situ* with the “shark-eye” camera (Fig. 9).

### Phylogenetic Reconstruction

**Figure 13:**
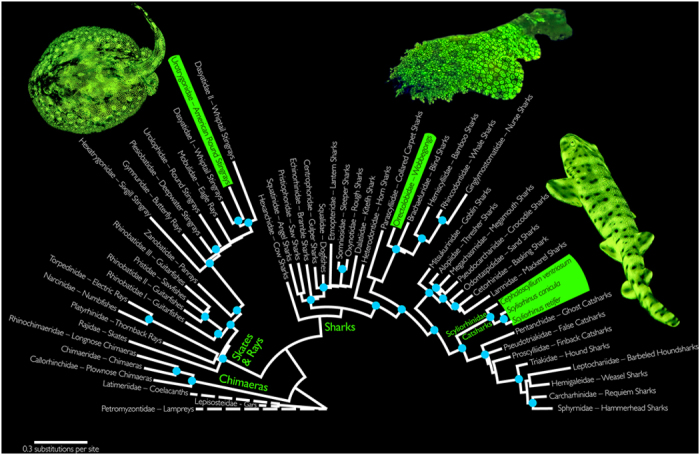
Family-level maximum likelihood phylogeny of elasmobranchs (species level phylogeny presented in Supp. Fig. 5). Blue circles on nodes indicate bootstrap support values ≥70%. Representatives of the three known biofluorescent elasmobranch clades are highlighted in green. Outgroups are marked with dashed lines. Image of biofluorescent orectolobid © BioPixel.

We conducted a molecular phylogenetic analysis using both mitochondrial and nuclear gene sequences to determine the distribution of biofluorescence in Elasmobranchii, as well as to determine the number of times the phenomenon has evolved in this diverse lineage. Our results show that biofluorescence has evolved at least three times in Elasmobranchii, in the distantly related families Urotrygonidae (American round stingrays), Orectolobidae (wobbegongs), and Scyliorhinidae (catsharks) (Fig. 13).

## Discussion

Light from the sun is quickly absorbed in the ocean, resulting in a stable narrowband blue spectral setting. This is an ideal environment for marine organisms to evolve biofluorescent compounds that absorb abundant blue, high-energy, short wavelength photons, which they emit back at longer, lesser energy wavelengths^21^. Following the recent report of biofluorescence in two species of catsharks, *C. ventriosum* and *S. retifer*^4^, we addressed the possibility that this spectral property plays a role in the biology and ecology of these small reclusive sharks.

With reference to the human visual system, biofluorescence had the effect of increasing color and brightness contrast (ratio of the perceived intensity of light), and therefore increasing the visibility of the sharks. There is often a tendency to describe animal coloration and pigmentation pattern from the perspective of the human visual system^31^,^32^. This is problematic because not only is color a sensation defined relative to the human visual system, but also the number of photoreceptors in the human eye and their spectral sensitivities differ greatly from most animals, especially those that live in aquatic habitats^33^. Thus, we first investigated whether these sharks could even detect the green fluorescent light being emitted by their conspecifics and found that both species could see well in this region of the visual spectrum. As would be expected for animals adapted for visual tasks in deep blue oceanic waters^34^, the individual visual pigment (i.e., each species has only one) in the eyes of *C. ventriosum* and *S. retifer* peaks at 484 ± 3 nm and 488 ± 5 nm (Fig. 1), respectively. These two species have pure rod retinas based on outer segment appearance. This condition has been found for several other elasmobranchs^35^,^36^,^37^,^38^; however, most other elasmobranchs have duplex retinas with both rods and cones similar to the vast majority of diurnal aquatic animals^39^.

The two species of catshark studied here possess essentially monochromatic vision typical of nocturnal or deep-dwelling species, which is intriguing as their visual pigment is slightly green-shifted compared to the 465 nm spectral average of deep ocean water^11^. *Cephaloscyllium ventriosum* occurs from relatively shallow habitats, down to over 360 m^40^, and *S. retifer* is found between ~70–550 m^41^. The deeper environments where these catsharks occur are dominated by the higher-energy, blue photons. *Cephaloscyllium ventriosum* is considered nocturnal and generally solitary, feeding at night by ambushing prey in a “lie and wait” fashion^42^. In this study, we observed *C. ventriosum* during the day and at night resting in groups of 2–5 individuals in crevices on the walls of Scripps Canyon and among rocks and kelp, as well as over sandy areas in the vicinity of Santa Barbara.

As a result, we wanted to determine whether biofluorescence provided these sharks an advantage in terms of visibility to conspecifics in their respective habitats. This led us to also perform hyperspectral analysis of the sharks’ skin to determine the precise wavelengths emitted by the fluorescent material in the dermis. Both sharks produced primarily green fluorescence (525–550 nm), although some blue fluorescence was also observed, especially for *C. ventriosum* when excited at 355 nm in the UV portion of the spectrum, which is thought to be a wavelength that has little ecological relevance given its poor penetration of the water column., The green spectral range of color produced by fluorescence of the shark’s skin corresponds to the dominant ambient light color calculated by the downwelling irradiance at shallow depths.

### Biofluorescence Enhances Luminosity Contrast with the Surrounding Marine Environment

**Figure 14:**
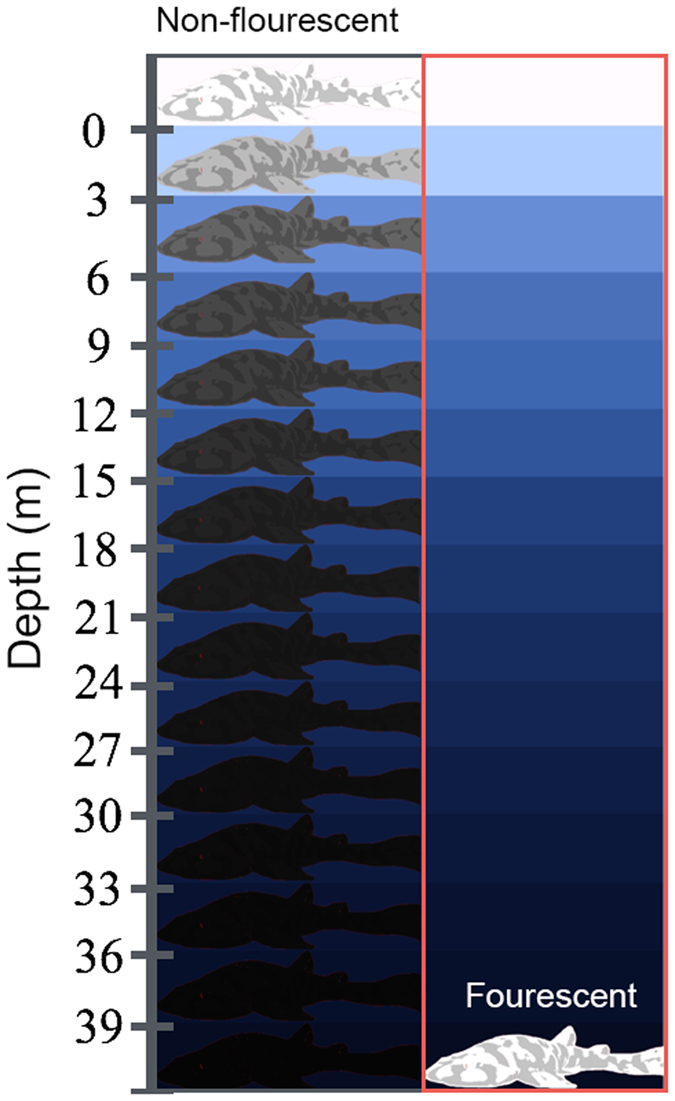
Heuristic illustration of the effect of fluorescence on the appearance of the swell shark, simulated from the perspective of other swell sharks. The left column shows the appearance of the shark based on the reflectance spectra of the skin and ambient light; the right column shows how the appearance changes when fluorescence is emitted due to excitation by narrowband light. The blue colors in each box are the RGB renderings of ambient light at that depth simulated using *in situ* irradiance measurements^65^ from Eilat, Israel (the most comprehensive published depth-gradient spectral data currently available). The white and gray patches in each shark drawing are scaled to show relative contrast rather than absolute colors. This illustration demonstrates that if the patches of the shark skin did not fluoresce, the contrast between the light and dark patches diminish with depth and the shark would match the background at depth. Fluorescence increases contrast between the dark/light patches of the shark by providing light spectra not naturally present in the blue ocean environment.

To quantify how much contrast changes with biofluorescence, we modeled the shark skin appearance under white light when it did not fluoresce and under narrowband light that excited fluorescence. How colors are perceived by the organisms observing them depends upon the spectrum of the ambient light in their habitat, the optical properties of the environment in which relevant visual tasks are performed, and their visual systems^43^. This is the first study to investigate the spectral sensitivities of the eyes of these sharks, revealing that they are monochromats most sensitive to light around 480 nm. No data on how these sharks perceive light exists. Thus, we used color perception tools developed for the human visual system and modeled sharks as monochromatic humans. We found that fluorescence increased the contrast between the gray and beige patches of the shark skin for both species. This trend was also captured by the “shark-eye” camera for *C. ventriosum* in its natural habitat. In essence, the luminosity contrast created by the shark’s biofluorescence can result in a conspecific being more apparent than a non-fluorescent shark (Fig. 14). The bright fluorescent “spots” on *C. ventriosum* would also correspond to anatomical areas of enhanced luminosity contrast (Fig. 4).

According to the “sensitivity hypothesis” for maximal discrimination of targets against a background based on brightness (luminance), a visual pigment matching the background spacelight is optimal^44^. Since the area under the absorbance spectrum of the visual pigment is the capture area, selection would place the visual pigment λ_max_ such that the absorbance envelope maximizes the overlap with the water spectral radiance in the direction of view. For clear blue water, as depth increases the spectral irradiance narrows with a peak in the blue, while overall brightness also decreases. For horizontal visual tasks, the sharks would be able to operate at great depth and still be above the signal-to-noise limit (see calculations of^45^). The 484 nm and 488 nm visual pigments found in *C. ventriosum* and *S. retifer,* respectively, are generally referred to as “deep-sea” visual pigments. They are spectrally situated to maximize quantum catch in clear blue oceanic water and are suited for carrying out visual tasks at depth. With only a single visual pigment and no pre-retinal filters, the task of detecting the fluorescent pattern against the background (non-fluorescing areas of skin or the background spacelight) can only utilize brightness (luminosity) differences. While the sharks 484 nm and 488 nm visual pigments are not spectrally situated to maximize contrast between the green fluorescence and the blue background spacelight, their visual pigment absorbance still overlaps the spectral bandwidth of the fluorescence which means that the dark and light reticulated patterns can still be detected.

These two fluorescent species of catshark live in deeper marine habitats subject to mainly blue light, and for which their visual pigments are adapted. *Cephaloscyllium ventriosum* was chosen for examination *in situ*, as opposed to *S. retifer*, as the latter species has a considerably deeper distribution (>73 m) and has not yet been observed directly via SCUBA^41^. The depth of ~30 m at which *C. ventriosum* was imaged *in situ* is the upper range of its habitat. Yet, at that depth, the shark is also able to see and identify conspecifics given that its λ_1/2max_ extends up to 540 nm.

### Phylogenetic Investigations Indicate that Biofluorescence Has Evolved at Least Three Times in Cartilaginous Fishes

**Figure 15:**
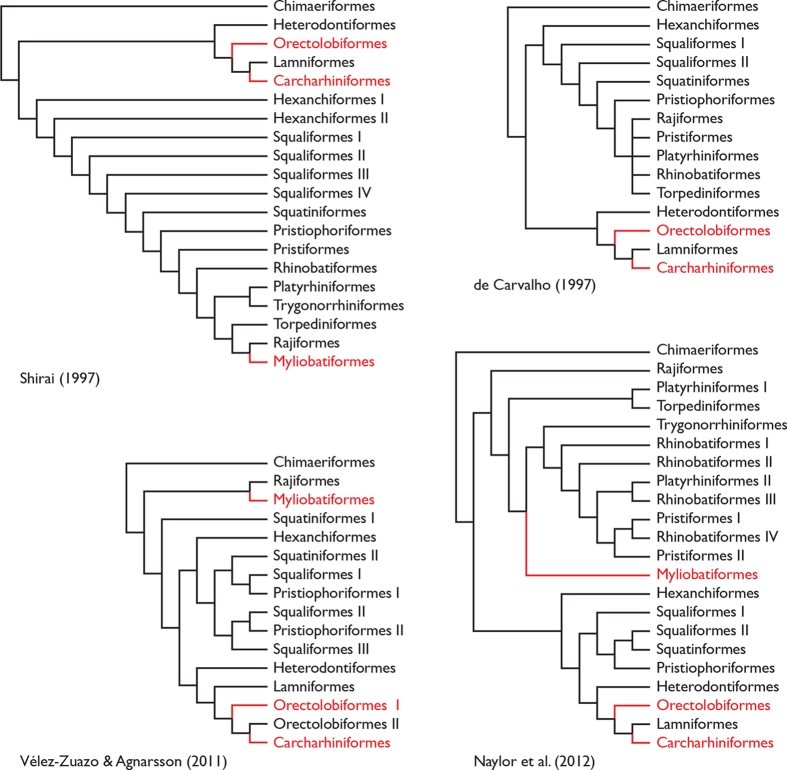
Previous phylogenetic hypotheses of elasmobranchs based on morphological data^46^,^47^ or DNA-sequence data^48^,^49^ highlighting the evolution of biofluorescence (red). In many cases the relevant biofluorescent families or genera were not included in these analyses, so optimizations are based on ordinal level presence only.

At present, there are currently five known elasmobranch species that exhibit green fluorescence. To lay the foundation and produce a predictive framework for understanding the evolution of biofluorescence in elasmobranchs, we generated a new phylogenetic hypothesis of the assemblage because previous studies (Fig. 15) either did not explicitly analyze all biofluorescent families^46^,^47^,^48^, or did not utilize more than a single mitochondrial gene for its phylogenetic reconstruction^49^. Further, these prior studies exhibit conflicting hypotheses of relationships among elasmobranchs, particularly with regard to the placement of batoids. Our novel phylogenetic hypothesis with more balanced sampling is more consistent with recent classifications (Supp. Table 1); only the Rhinobatiformes were recovered as polyphyletic. Beyond its utility in exploring our current understanding of the evolution of biofluorescence in elasmobranchs, the resulting phylogenetic hypothesis presents a multi-gene hypothesis of relationships for elasmobranchs that researchers can use as a predictive framework for exploring the evolution of this largely unexplored phenomenon^4^,^50^. Further, this novel phylogenetic framework will aid researchers exploring the evolution of the compounds involved in elasmobranch fluorescence.

The five known fluorescent elasmobranch species belong to three distantly related families, Urotrygonidae (American round stingrays), Orectolobidae (wobbegongs), and Scyliorhinidae (catsharks), indicating that biofluorescence has evolved at least three times in elasmobranchs (Fig. 13). For bony fishes, we reported that biofluorescence was most common and interspecifically distinct in cryptically patterned, reclusive coral reef species^4^. Interestingly, these three elasmobranch families also comprise reclusive and cryptically patterned species that are difficult to spot in their natural environment; and perhaps for these reasons, the predators of these sharks are not known. We caution that our sampling of biofluorescence in lineages within Elasmobranchii has not been exhaustive, and it is likely that biofluorescence has evolved in additional families and will be even more widespread in sharks and rays than we currently know (Fig. 13). We have been been able to scan only a few holocephalans (chimaeras) to date; however, fluorescence has not yet been detected in this lineage. Our recognition that biofluorescence is most prevalent in cryptic reef fishes, combined with the predictive phylogenetic hypothesis, identifies the families that should be explored in more detail or that require even an initial investigation of this feature.

Although biofluorescence is herein shown to be phylogenetically widespread in elasmobranch fishes, occurring in three distantly related lineages, our current investigation suggests that this characteristic has not evolved nearly as many times as we observe in the considerably more diverse bony fishes. In contrast to the biofluorescent representatives in Orectolobidae, Scyliorhinidae, and Urotrygonidae, the examined representatives from Carcharhiniformes (Carcharhinidae, Sphyrnidae, Triakidae), Heterodontiformes (Heterodontidae), Lamniformes (Odontaspididae), Myliobatiformes (Dasyatidae, Myliobatidae), Orectolobiformes (Ginglymostomatidae), Squaliformes (Squalidae), and Torpediniformes (Torpedinidae) were not observed to be biofluorescent.

Scyliorhinids are enigmatic elasmobranch species that live in shallow to deep environments. Scyliorhinidae is a moderately large family that comprises nine genera and 66 species^51^. *Cephaloscyllium ventriosum* is one of 18 currently recognized species of *Cephaloscyllium*^52^ and is endemic to the eastern Pacific^53^. In this study, we examined the two known fluorescent species of catsharks (Scyliorhinidae), although it is quite likely that with further study, several additional (or all) members of this family will be shown to exhibit green fluorescence. In both species, fluorescent materials arranged in distinct patterns are located solely on the dermis. In *C. ventriosum* these patterns appear to be sexually dimorphic with females having a unique “face mask” and more dense ventral spotting that extends further anteriorly than in males. In addition, the pelvic claspers of males, which are lacking in females, are strongly fluorescent in both *C. ventriosum* and *S. retifer* (Fig. 5). Sexual dimorphism in the dermal denticles has also previously been reported for the Lesser-Spotted Catshark (*Scyliorhinus canicula*)^54^.

Given the widespread, but not ubiquitous, presence of biofluorescence in elasmobranchs, coupled with a visual apparatus to detect the phenomenon and the potential for fluorescent skin to create greater contrast at depth, leads to questions regarding the distribution and evolution of biofluorescence in cartilaginous fishes in general and the role fluorescence plays in shark and ray behavior, biology, and diversification. The fact that these cryptically patterned biofluorescent sharks and rays are capable of visualizing their own fluorescent emissions, coupled with sexually dimorphic fluorescent patterns and the fact that they would be difficult to spot by predators lacking similar visual capabilities, is suggestive of a communication/species recognition role for fluorescence. Biofluorescence could also potentially help camouflage catsharks against potential predators who do have color vision^55^, although very little is known regarding the predators of catsharks and the visual systems of only a few marine organisms have been studied in detail. Round stingrays (e.g., *Urobatis jamaicensis*) that emit green fluorescence^4^ possess three cone visual pigments (475 nm, 533 nm, and 562 nm) and two rod pigments (500 nm and 499 nm)^6^, which indicates sensitivity to wavelengths in their biofluorescent emission range. Biofluorescent footage of the wobbegong or carpet sharks (Orectolobidae) has also been documented by Richard Fitzpatrick of Biopixel (unpublished).

Historically, the visual sense of elasmobranchs has been a topic of investigation for over a century^56^, although considered of minor importance compared with their other senses, particularly olfaction. It is now known that many elasmobranchs possess well-developed eyes^36^, as well a large sensory brain dedicated to processing visual information^36^. Yet, the visual system of just a few of the nearly 1,235 elasmobranch species has been investigated in detail (see^9^, for review). Catsharks possess the ability to detect the green biofluorescence that is emitted by their conspecifics and this fluorescence creates greater contrast with the surrounding habitat in deeper blue-shifted waters (under solar or lunar illumination).

## Methods

### Microspectrophotometry

*Cephaloscyllium ventriosum* was obtained from Marinus Scientific, LLC, and *S. retifer* was obtained from the Riverhead Aquarium (NY). This study was approved and carried out in strict accordance with the recommendations in the Guidelines for the Use of Fishes in Research of the American Fisheries Society and Cornell University’s Institutional Animal Care and Use Committee (IACUC). Specimens were examined within 4 hr of their arrival at Cornell University. Specimens were dark adapted for at least 2 hr at 50 °C to facilitate handling prior to sacrifice by pharmaceutical-grade MS-222 (IACUC protocol # 2014-0112). Enucleations were performed under dim red light (Kodak safelight No. 2, 15 W bulb), with all further preparations done under infrared (IR) lighting using LEDs and appropriate image converters. Eyes were hemisected and placed in cold phosphate buffer, pH 7.4, supplemented with 6% sucrose. A small piece of the isolated retina was transferred to a cover slip where it was macerated with razor blades and sealed with an overlying cover slip edged with silicone grease.

Microspectrophotometry (MSP) was conducted using a computer-controlled, single-beam instrument, previously described^57^, that classifies visual pigments based on their distinct spectral absorbance characteristics and measures spectral sensitivities, and hence determines the performance and spectral limits of vision. To enable measurement into the UV, a Zeiss 32× 0.4 NA Ultrafluar was used as the condenser and a 100× 1.0 NA LOMO UV lens was used as the objective. With a 100 W tungsten-halogen lamp, it was possible to measure down to 350 nm with excellent signal to noise ratio and minimal visual pigment bleaching. All spectra ultimately saved and analyzed consisted of the even nanometer data from the down-scan interleaved with the odd nanometer data from the return scan. A Labview (National Instruments, Austin, TX) program was developed to handle all scanning, baseline correction, data acquisition, and file storage. A separate Labview program was used for data analysis. A Gaussian function was fit to the top 40 raw data points on either side of the estimated peak and an x_max_ determined by differentiation. The data were then normalized. Both vitamin A1- and A2-based visual pigment template curves using the calculated x_max_ were constructed based on^58^. The λ_max_ was found by ‘sliding’ the templates along the wavelength axis and obtaining the best fit to the raw data as determined by ‘least squares’ fitting. The λ_max_ obtained was verified by visual examination.

### Hyperspectral Imaging

*Cephaloscyllium ventriosum* was assessed under a hyperspectral imager in fluorescence mode (using the PARISS^®^ technology; LightForm Inc., Asheville, NC). Areas of skin (0.5 × 0.5 cm; 10× objective) obtained from the flat dorsal part of the head anterior to the dorsal fin, were mapped directly from the specimen. Using hyperspectral mapping analysis, each spectrum is acquired in full instantaneously, with <2 nm resolution (best between 380–950 nm). The technique thus provides a spatial dimension to spectra and allows for the assessment of morphological or ultra-structural distribution of spectra. Similar skin areas were also photographed in fluorescence under a Nikon 80i microscope using a monochrome Retiga 2000DC, CCD camera (QImaging, BC, Canada). Skin areas always included one of the darker/black spots (in bright field) separated by light beige areas, typical for these sharks. Wavelength calibrations are performed with a MIDL Hg+/Ar+ emission lamp (LightForm, Inc.). For relative comparison of spectra intensity, all acquisition parameters were kept the same, except for exposure time, which can be corrected for afterwards. All spectra are expressed relative to the lowest exposure time used across samples. Spectra from one individual sample showing >99% closeness of fit are identified as a single representative spectrum.

### *In-situ* Imaging

Individuals of *C. ventriosum* were observed and imaged along the walls of Scripps Canyon, San Diego County, between 90–150 feet depth, in October 2014 and January 2015. A Red Epic-M 4K camera (Red Digital Cinema, Irvine, CA) mounted in an Aquatica Rouge underwater housing was used for three different kinds of imaging: white light, fluorescent, and “shark-eye”.

#### White light imaging

A Nikon NF-S Nikkor 24 mm 1:1.4G ED lens was used to image the sharks during the day under natural light using the setup described above.

#### Fluorescence imaging

To excite a fluorescence response, the Aquatica Rouge housing was fitted with custom designed blue excitation lighting. The LED light (Royal Blue) was collimated to ensure its perpendicular incidence on the scientific grade 450/70 nm interference filter surface (Semrock, Inc., Lake Forest, IL), minimizing the transmission of out-of-band energy. The ultra-bright LEDs, collimating lenses, filters, and exit diffusers were contained in custom-made water- and pressure-proof housings and powered by NiMH Battery Packs (Ikelite Underwater Systems, Indianapolis, IN). To image and record biofluorescence, a scientific-grade 514 nm long-pass emission filter (Semrock, Inc.) was embedded in front of the sensor of the camera. Cross-sections of both sharks were taken on an AxioZoom v16 stereo fluorescent microscope using a PlanNeoFluar Z 2.3×/0.57 objective and 38 HE GFP filter set.

#### Shark-eye imaging

To simulate the spectral response of the eye of *C. ventriosum* we devised a “shark-eye” camera that comprises a Red Epic camera, a pack consisting of a Wratten 44 A gelatin filter (Eastman Kodak, Rochester, NY), a 575 nm shortpass filter, and a 400 nm longpass filter (Edmund Optics, Inc., Barrington, NJ) placed over the Nikon NF-S Nikkor 24 mm 1:1.4G ED lens (Fig. 8). Imaging was conducted under natural light conditions. Camera spectral response specifications were obtained from^59^, and filter specifications were extracted from www.edmundoptics.com using the Data Thief software^60^. Since the spectral responses of the two catshark species examined are similar, and *S. retifer* is found in deep waters beyond the range of SCUBA, “shark-eye” imaging was only simulated for *C. ventriosum*.

#### RGB simulation of shark appearance

Given that this is the first study that presents the photoreceptor sensitivities of *S. retifer* and *C. ventriosum*, psychophysics-based models of their lightness perception do not exist. Therefore, to gain some insight regarding the appearance of these sharks to conspecifics when emitting fluorescence, as opposed to their appearance under broadband daylight, we employed the CIE 1931 XYZ color space^61^ that was developed to represent color perception for a human with trichromatic vision. Using this color space and the CIE 1931 2° Standard Observer Curves, we modeled the shark eye as a monochromatic human with the 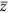 spectral sensitivity curves given in Fig. 1. We use the reflectance spectra for dark and beige skin components of each shark species from Fig. 2 taken under white light and emitted after exposure to 470 nm monochromatic light, respectively, and for simplicity assume an ambient light profile of uniform white light. Radiance and fluorescence spectra for dark and beige skin components of each shark species were converted into XYZ tri-stimulus values and then to sRGB color space^30^. For simplicity of viewing and visual comparison, the intensity of the beige patches in an image of *C. ventriosum* (Fig. 10) and *S. retifer* (Fig. 11) has been adjusted to be white (RGB = [255,255,255]), and the ratio of the intensity of the beige patch to the dark patches are inscribed in the top left corner of each image. The appearance of the sharks to the “shark-eye” camera was also modeled using the trichromatic response of the camera sensor from Fig. 9, and its monochromatic version by only taking the blue-channel response.

### Modeling of Visual Pigments

A model^29^ was employed to calculate what visual pigment(s) would be best at discriminating green fluorescence and some gray targets based on luminosity contrast using the background spacelight estimated for increasing depths in Scripps Canyon (San Diego County, California). A second analysis was conducted to determine what water color is a best match for the 484 nm (*C. ventriosum*) and 488 nm (*S. retifer*) visual pigments, along with the best pigments for discriminating gray targets in clear, blue oceanic water (i.e., no chlorophyll or dissolved organic matter). At some point, the contrast between the ‘target’ and the background, or other targets, reaches a limit set by the signal-to-noise ratio of the photoreceptors that contain a particular visual pigment. Although the model can deal with chromatic contrast situations, the fact that only a single visual pigment was found limited our use of the model to luminosity contrast alone. The model was also used to calculate the water color that would maximize the contrast for a visual system with a single visual pigment (484 nm for *C. ventriosum* and 488 nm of *S. retifer*) when observing gray targets, or a target with a spectral radiance like that of the fluorescent patches.

### Phylogeny Reconstruction

To explore the evolution of biofluorescence in elasmobranchs, 77 taxa were analyzed in the current study, including 74 chondrichthyans representing 51 cartilaginous families. The GenBank sequences for the analyzed species can be found in Supp. Table 1. Alignments were generated using MUSCLE^62^, with the default parameters and a maximum number of iterations of ten. For the phylogenetic analysis, a total of 4,169 nucleotides from three genes, ND2 (1,047 base pairs), COI (647 base pairs), and RAG 1 (2,475 base pairs), were included. The dataset was partitioned by both individual gene fragments and codon position within each gene fragment for a total of nine partitions. A model of molecular evolution was chosen by the program jMODELTEST v.2.1^63^, with the best fitting model under the Akaike information criteria (AIC) for each individual gene partition assigned, including: ND2 (1st position: GTR+I+G, 2nd position: GTR+I+G, 3rd position: TVM+I+G), COI (1st position: GTR+I+G, 2nd position: JC, 3rd position: HKY+G), and RAG1 (1st position: GTR+I+G, 2nd position: GTR+I+G, 3rd position: SYM+G). The maximum likelihood analysis was conducted in GARLI v2.01^64^, and the tree with the best likelihood score from 100 independent analyses was selected as the preferred hypothesis. A nonparametric maximum-likelihood bootstrap analysis was conducted for 200 random pseudoreplicates to assess nodal support. The tree with the optimal likelihood score is presented here (Fig. 13 and Supp. Fig. 5) to evaluate the evolutionary relationships of cartilaginous fishes and explore the distribution and diversity of biofluorescence.

## Additional Information

**How to cite this article**: Gruber, D. F. *et al*. Biofluorescence in Catsharks (Scyliorhinidae): Fundamental Description and Relevance for Elasmobranch Visual Ecology. *Sci. Rep.* **6**, 24751; doi: 10.1038/srep24751 (2016).

## Supplementary Material

Supplementary Information
