# Supplementary material for: Biofluorescence in Catsharks (Scyliorhinidae): Fundamental Description and Relevance for Elasmobranch Visual Ecology
Source: Sci Rep. 2016 Apr 25;6:24751. doi: 10.1038/srep24751 (PMC4843165; doi:10.1038/srep24751)
Supplement: Supplementary Information [file srep24751-s1.pdf]

Supplementary Table and Figures

Biofluorescence in Catsharks (Scyliorhinidae): Fundamental Description and Relevance for Elasmobranch Visual Ecology

David F. Gruber[1,2*], Ellis R. Loew[3], Dimitri D. Deheyn[4], Derya Akkaynak[5], Jean P. Gaffney[1], W. Leo Smith[6], Matthew P. Davis[7], Jennifer H. Stern[6], Vincent A. Pieribone[8], John S. Sparks[9]

[1]Baruch College
City University of New York
Department of Natural Sciences
New York, NY 10010, USA

[2]City University of New York
The Graduate Center
Program in Biology
New York, NY 10016, USA

[3]College of Veterinary Medicine
Cornell University
Ithaca, NY 14853, USA

[4]University of California, San Diego
Scripps Institution of Oceanography
La Jolla, CA 92093, USA

[5]University of Haifa
Charney School of Marine Sciences
Haifa, 3498838, Israel
Interuniversity Institute of Marine Sciences
Eilat, 88103, Israel

[6]University of Kansas
Biodiversity Institute and Department of Ecology and Evolutionary Biology
Lawrence, KS, 66049 USA

[7]St. Cloud State University
Department of Biological Sciences
St. Cloud, MN 56301 USA

[8]The John B. Pierce Laboratory
Cellular and Molecular Physiology
Yale University School of Medicine
New Haven, CT 06519, USA

[9]American Museum of Natural History
Division of Vertebrate Zoology

Supplementary Information

Supp. Table 1. Classification and GenBank accession numbers for species included in phylogenetic analyses.

Supp. Fig. 1. *Cephaloscyllium ventriosum* in its natural environment in Scripps Canyon under natural light conditions, with no filter or added light.

Supp. Fig. 2. Top) Cross section of *C. ventriosum* showing localization of fluorescent compound in the "lighter" areas of the skin and in basal layer of dermis under white light (top left) and fluorescent light (top right). Bottom) 150X magnification of a single dermal dendrite showing fluorescence at base of dermal dendrite. White light (bottom left), fluorescent light (bottom right).

Supp. Fig. 3. Top) Cross section of *S. retifer* showing localization of fluorescent compound in basal layer of dermis.

Supp. Fig. 4. Fluorescence exhibited in egg casings of *S. retifer* at different life-stages.

Supp. Fig. 5. Species level maximum likelihood phylogeny of elasmobranchs. Numbers at nodes indicate bootstrap support values ≥50%. Asterisks (*) at nodes indicate bootstrap support values ≥95%. Outgroups marked with dashed lines.

Supplementary Table 1. Classification and GenBank accession numbers for species included in phylogenetic analyses.

TAXON	RAG1	ND2	COI
**PETROMYZONTIFORMES**			
Petromyzon_marinus	//	U11880.1	U11880.1
**COELACANTHIFORMES**			
**Coelacanthidae**			
Latimeria_menadoensis	AY442925.1	NC_006921.2	NC_006921.2
**LEPISOSTEIFORMES**			
**Lepisosteidae**			
Lepisosteus_osseus	//	NC_008104.1	NC_008104.1
**CHIMAERIFORMES**			
**Callorhinchidae**			
Callorhinchus_milii	//	HM147137.1	HM147137.1
**Chimaeridae**			
Chimera_sp	GU130744.1	//	GU130671.1
Hydrolagus_colliei	//	JQ518720.1	JQ354134.1
**Rhinochimaeridae**			
Rhinochimaera_pacifica	//	HM147141.1	HM147141.1
**HEXANCHIFORMES**			
**Hexanchidae**			
Heptranchias_perlo	//	KF927891.1	KF590398.1
**SQUALIFORMES**			
**Dalatiidae**			
Squaliolus_aliae	GU130748.1	KF927966.1	GU130675.1
Dalatias_licha	GU130749.1	JQ518951.1	GU130676.1
Isistius_brasiliensis	GU130817.1	JQ518953.1	//
**Etmopteridae**			
Etmopterus_lucifer	GU130785.1	JQ518963.1	GU130682.1
Etmopterus_pusillus	GU130762.1	JQ518964.1	KF899426.1
Trigonognathus_kabeyai	GU130775.1	JQ400130.1	GU130702.1
**Somniosidae**			
Proscymnodon_plunketi	GU130769.1	KF927991.1	GU130696.1
**Oxynotidae**			
Oxynotus_paradoxus	GU130747.1	JQ518966.1	GU130674.1
**Centrophoridae**			
Deania_calcea	GU130772.1	JQ519007.1	DQ108225.1
Centrophorus_squamosus	GU130774.1	JQ035655.1	KF899383.1
**Squalidae**			

Squalus_acanthias	JN184093.1	JQ035621.1	KC015925.1
**Echinorhinidae**			
Echinorhinus_brucus	GU130818.1	JQ519170.1	HM467790.1
Echinorhinus_cookei	//	JQ519016.1	DQ521002.1
**PRISTIOPHORIFORMES**			
**Pristiophoridae**			
Pristiophorus_japonicus	//	AB721306.1	AB721306.1
**SQUATINIFORMES**			
**Squatinidae**			
Squatina nebulosa	//	KF927985.1	KM264313.1
**HETERODONTIFORMES**			
**Heterodontidae**			
Heterodontus_francisci	JN184089.1	JQ519165.1	FJ519566.1
**LAMNIFORMES**			
**Odontaspididae**			
Carcharias_taurus	AF135475	KF569943.1	KF569943.1
**Mitsukurinidae**			
Mitsukurina_owstoni	JN184090.1	EU528659.1	EU528659.1
**Pseudocarchariidae**			
Pseudocarcharias_kamoharai	AF135479.1	KM575726.1	KM575726.1
**Lamnidae**			
Lamna_ditropis	AF135478.1	JQ518735.1	KF918878.1
Isurus_oxyrinchus	AF135480.1	JQ518734.1	KF590330.1
**Megachasmidae**			
Megachasma_pelagios	AF135483.1	KC702506.1	KC702506.1
**Cetorhinidae**			
Cetorhinus_maximus	AY462147.1	KF597303.1	KF597303.1
**Alopiidae**			
Alopias_pelagicus	AF135473	KF412639.1	KF412639.1
**ORECTOLOBIFORMES**			
**Rhincodontidae**			
Rhincodon_typus	//	KC633221.1	KC633221.1
**Parascylliidae**			
Parascyllium_collare	//	JQ518749.1	//
**Brachaeluridae**			
Brachaelurus_colcloughi	//	JQ519055.1	//
**Orectolobidae**			

Orectolobus_japonicus	//	KF111729.1	KF111729.1
**Hemiscyllidae**			
Chiloscyllium_punctatum	//	JQ082337.1	JQ082337.1
**Ginglymostomatidae**			
Ginglymostoma_cirratum	AY949032.1	JQ518741.1	//
Nebrius_ferrugineus	//	JQ518742.1	KF899636.1
**CARCHARHINIFORMES**			
**Pentanchidae**			
Apristurus_melanoasper	AY462161.1	JQ519193.1	EU398537.1
**Scyliorhinidae**			
Cephaloscyllium_ventriosum	//	JQ519118.1	GU440268.1
Scyliorhinus_retifer	//	JQ519117.1	KT075309.1
Scyliorhinus_canicula	JN184092.1	NC_001950.1	NC_001950.1
**Proscylliidae**			
Proscyllium_habereri	AY462184.1	KF927940.1	JQ681382.1
**Pseudotriakidae**			
Pseudotriakis_microdon	AY462185.1	AB560493.1	AB560493.1
**Leptochariidae**			
Leptocharias_smithii	//	JQ518660.1	//
**Triakidae**			
Mustelus_asterias	AY462188.1	JQ518705.1	KJ709832.1
**Hemigaleidae**			
Hemipristis_elongata	//	JQ519069.1	KP193382.1
**Carcharhinidae**			
Carcharhinus_acronotus	//	GU385350.1	KF461148.1
Eusphyra_blochii	//	DQ422104.1	EU398784.1
**Sphyrnidae**			
Sphyrna_lewini	JQ929571.1	JQ519063.1	KF009669.1
**RAJIFORMES**			
**Rajidae**			
Leucoraja_naevus	JN184231.1	JN184280.1	KP975730.1
Okamejei_hollandi	//	JQ519059.1	JQ681338.1
**PLATYRHINIFORMES**			
**Platyrhinidae**			
Platyrhina_sinensis	JN184111.1	JN184068.1	HM180795.1
Platyrhinoidis_triseriata	JN184112.1	JQ518853.1	GU440463.1
**TORPEDINIFORMES**			
**Narcinidae**			

Narcine_tasmaniensis	JN184094.1	JN171594.1	JN171594.1
**Torpedinidae**			
Torpedo_macneilli	JN184095.1	JQ518927.1	EU399058.1
**TRYGONORRHINIFORMES**			
**Trygonorrhinidae**			
Trygonorrhina_fasciata	JN184102.1	JQ519027.1	EU399098.1
Zapteryx_exasperata	JN184103.1	KC610081.1	JN184087.1
**RHINOBATIFORMES**			
**Rhinobatidae**			
Anoxypristis_cuspidata	JN184212.1	JN184261.1	KF590517.1
Pristis_clavata	JN184097.1	JQ519150.1	EU398988.1
Rhinobatos_glaucostigma	JN184100.1	JQ518911.1	JN184075.1
Glaucostegus_thouin	JN184215.1	JN184264.1	KF899441.1
Glaucostegus typus	JN184098.1	JQ519057.1	EU398998.1
**Zanobatidae**			
Zanobatus_schoenleinii	JN184113.1	JQ518943.1	//
**Hexatrygonidae**			
Hexatrygon_bickelli	JN184120.1	JQ518835.1	JN184061.1
**Plesiobatidae**			
Plesiobatis_daviesi	JN184125.1	KF927935.1	EU398978.1
**Dasyatidae**			
Pastinachus_atrus	KC249808.1	JN184290.1	KM072988.1
Urogymnus_asperrimus	KC249812.1	JQ519107.1	KC250636.1
Himantura_walga	KC249807.1	JN184308.1	KM072995.1
Himantura leopardа	//	KM073045.1	JX263418.1
**Gymnuridae**			
Gymnura_crebripunctata	JN184119.1	JQ518827.1	JN184060.1
Gymnura_micrura	JN184244.1	JN184295.1	GU225294.1
Aetoplatea_zonura	JN184243.1	JN184294.1	EU398805.1
**Myliobatidae**			
Mobula_thurstoni	KM364956.1	KM396925.1	KM073012.1
**Urolophidae**			
Urolophus_cruciatus	JN184129.1	JQ518937.1	DQ108152.1
**Potamotrygonidae**			
Taeniura_lymma	KC249810.1	KM073038.1	KC250633.1
**Urotrygonidae**			
Urobatis_jamaicensis	//	JQ518941.1	GU225505.1

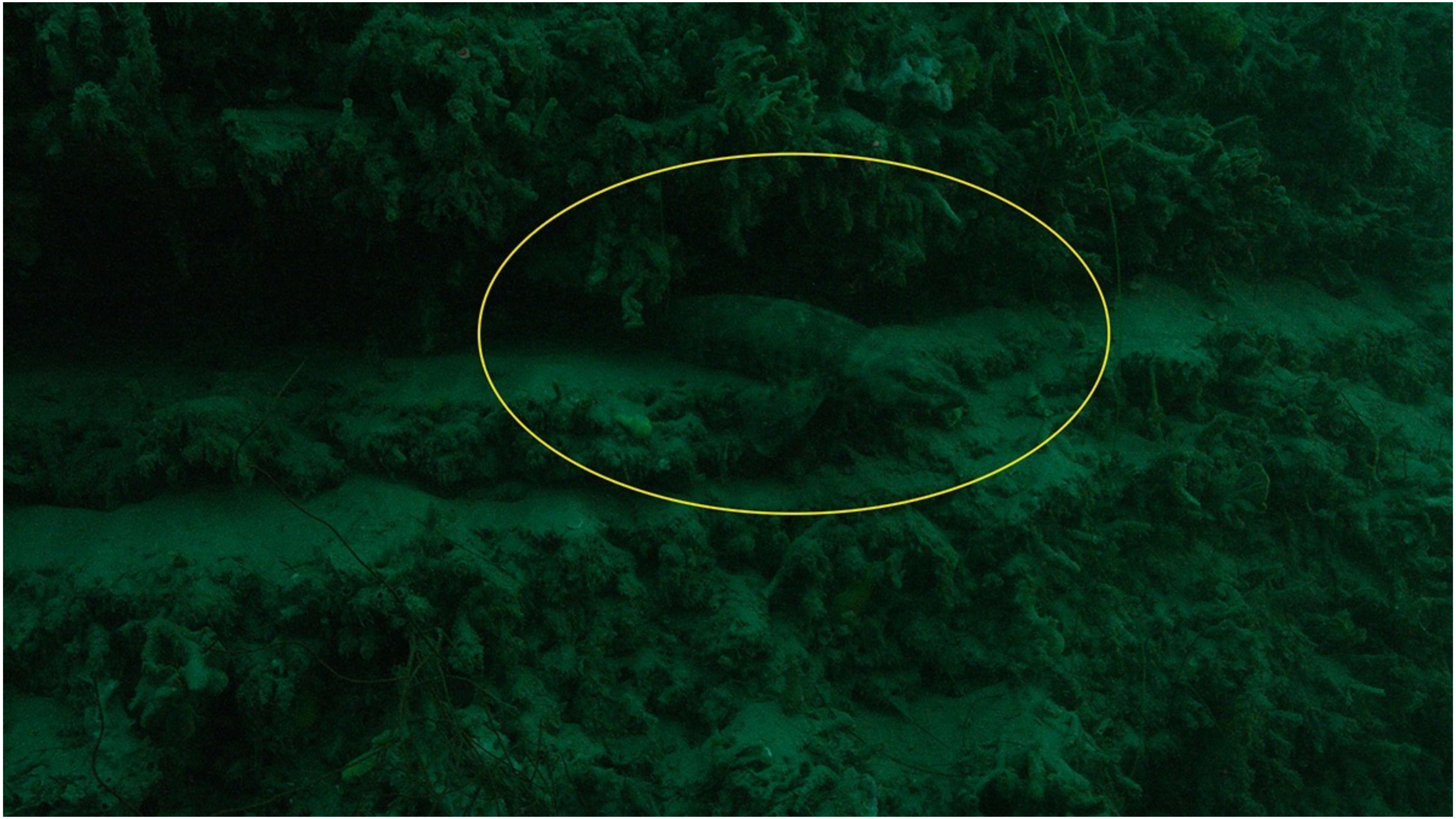

Supp. Fig. 1.

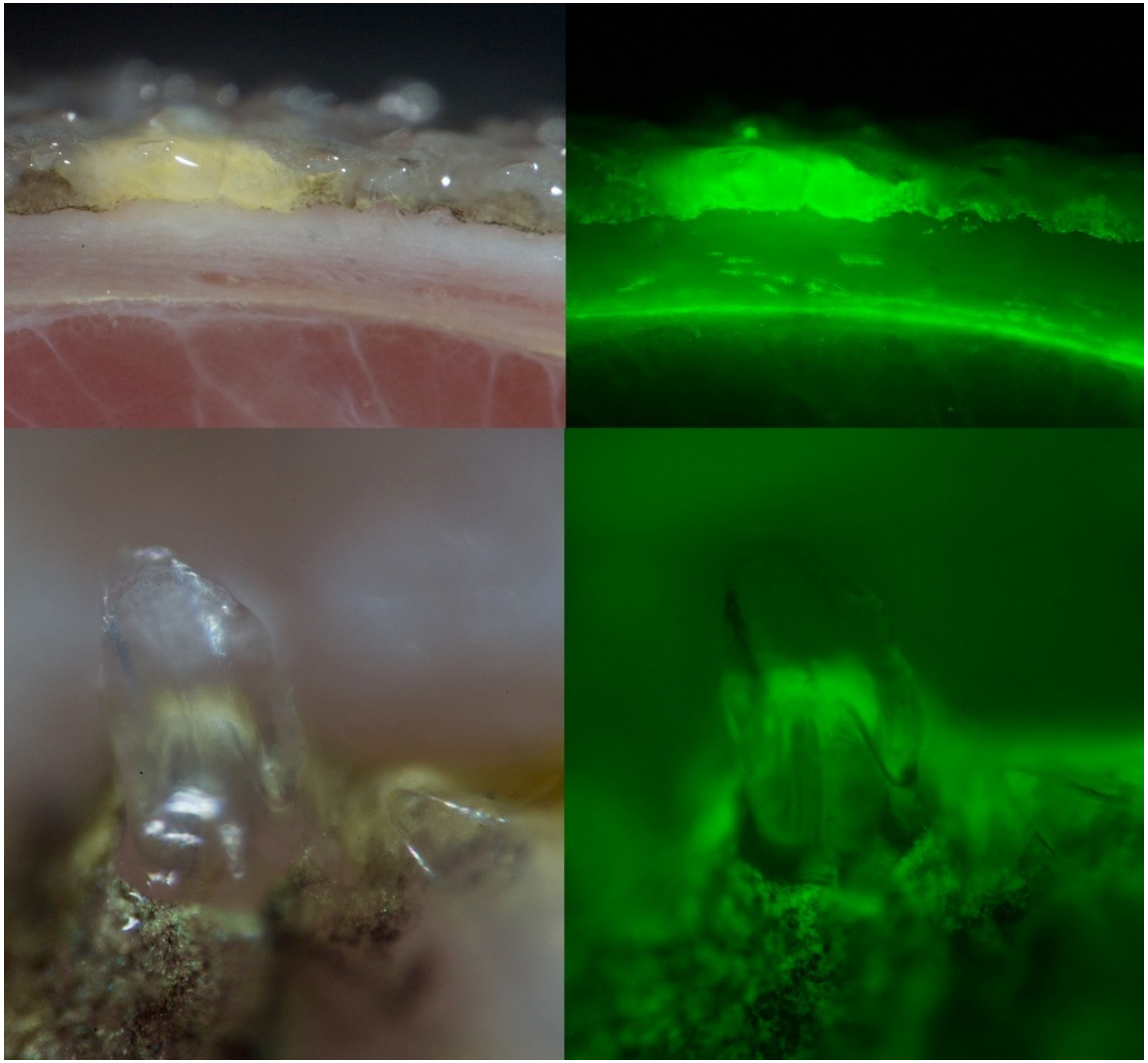

Supp. Fig. 2.

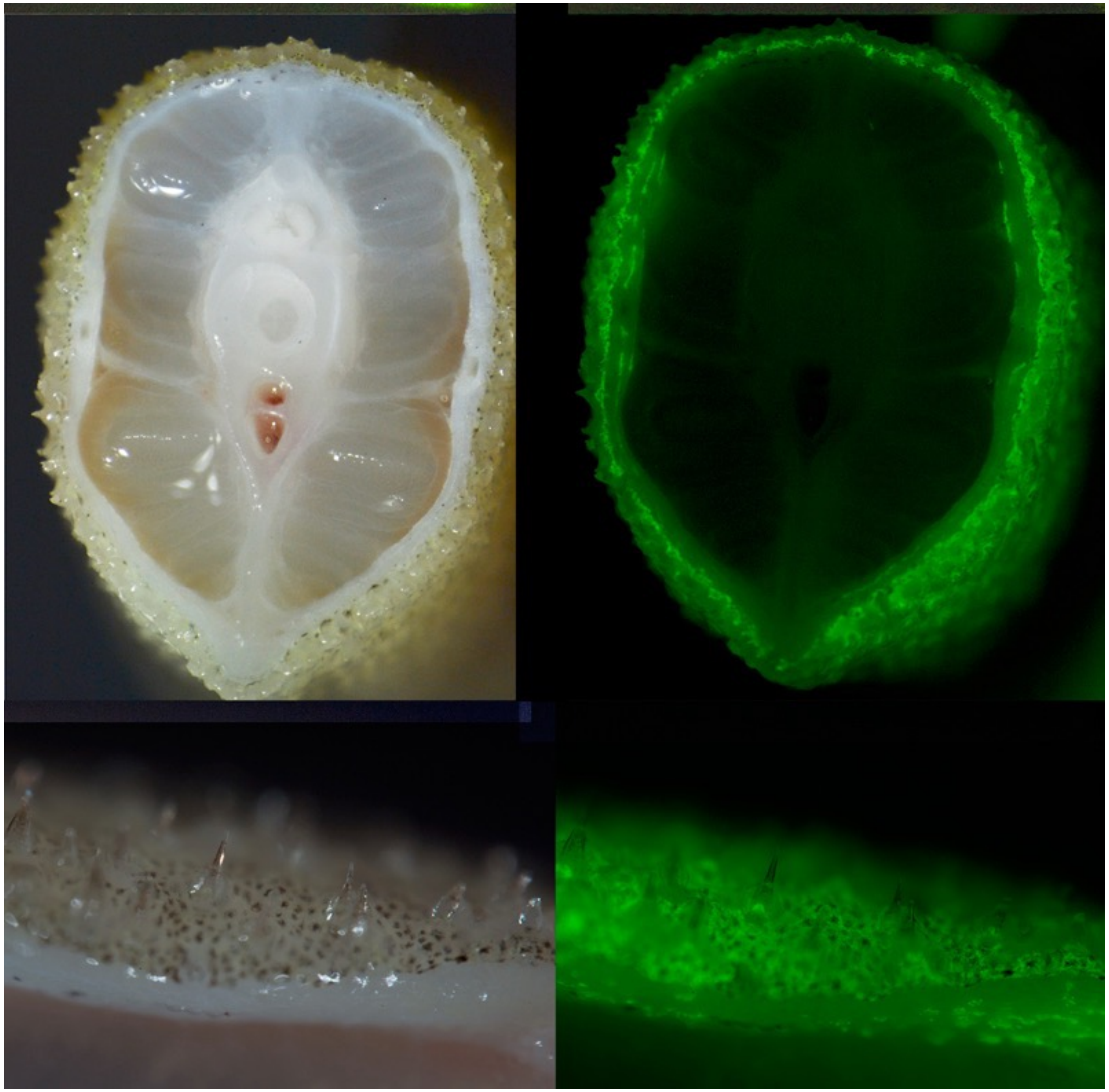

Supp. Fig. 3.

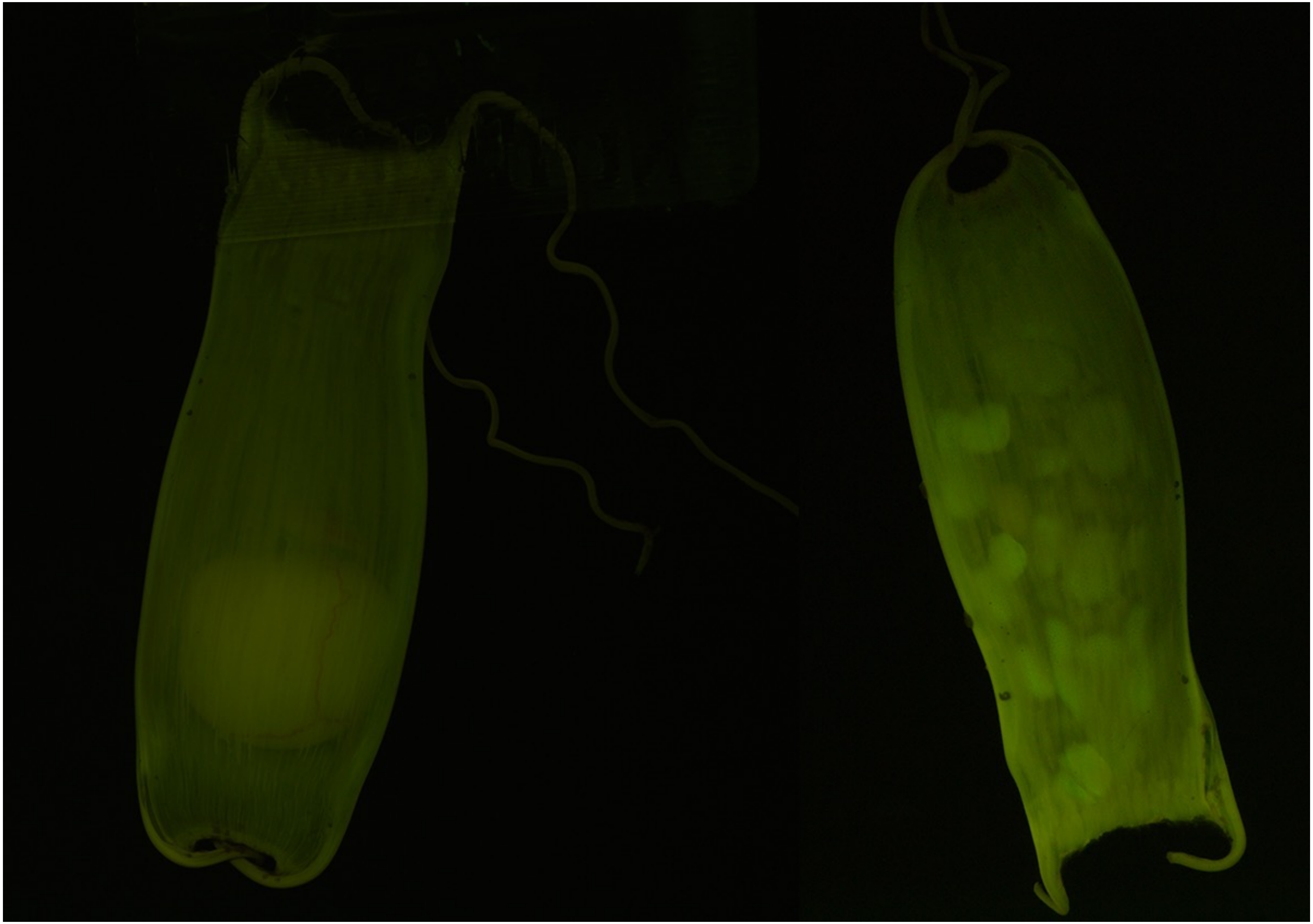

Supp. ***Fig. 4.***

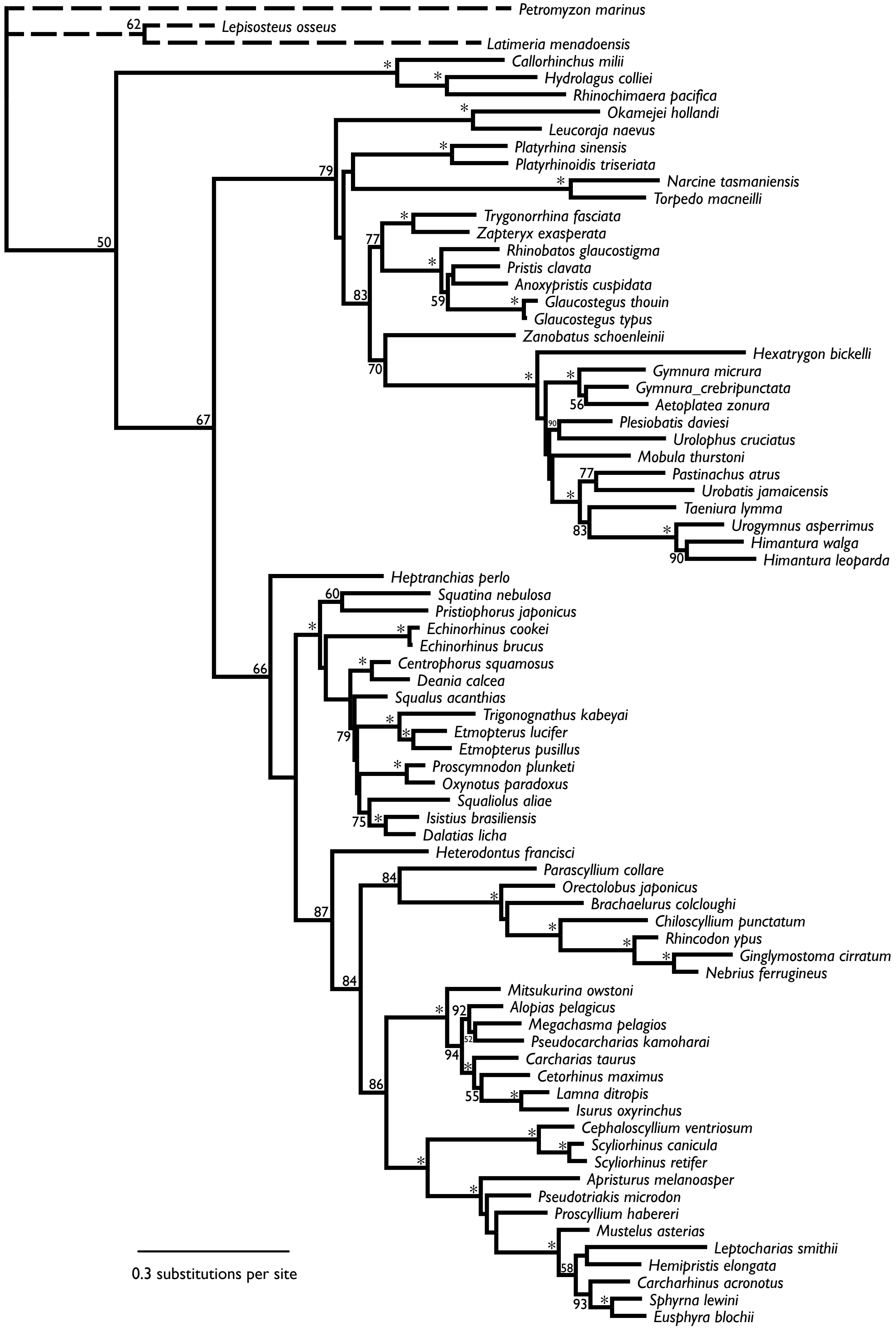
Petromyzon marinus
62
Lepisosteus osseus
Latimeria menadoensis
Callorhinchus milii
Hydrolagus colliei
Rhinochimaera pacifica
Okamejei hollandi
Leucoraja naevus
Platyrhina sinensis
Platyrhinoidis triseriata
Narcine tasmaniensis
Torpedo macneilli
Trygonorrhina fasciata
Zapteryx exasperata
Rhinobatos glaucostigma
Pristis clavata
Anoxypristis cuspidata
Glaucostegus thouin
Glaucostegus typus
Zanobatus schoenleinii
Hexatrygon bickelli
Gymnura micrura
Gymnura_crebripunctata
Aetoplatea zonura
Plesiobatis daviesi
Urolophus cruciatus
Mobula thurstoni
Pastinachus atrus
Urobatis jamaicensis
Taeniura lymma
Urogymnus asperrimus
Himantura walga
Himantura leoparda
Heptranchias perlo
Squatina nebulosa
Pristiophorus japonicus
Echinorhinus cookei
Echinorhinus brucus
Centrophorus squamosus
Deania calcea
Squalus acanthias
Trigonognathus kabeyai
Etmopterus lucifer
Etmopterus pusillus
Proscymnodon plunketi
Oxynotus paradoxus
Squaliolus aliae
Isistius brasiliensis
Dalatias licha
Heterodontus francisci
Parascyllium collare
Orectolobus japonicus
Brachaelurus colcloughi
Chiloscyllium punctatum
Rhincodon ypus
Ginglymostoma cirratum
Nebrius ferrugineus
Mitsukurina owstoni
Alopias pelagicus
Megachasma pelagios
Pseudocarcharias kamoharai
Carcharias taurus
Cetorhinus maximus
Lamna ditropis
Isurus oxyrinchus
Cephaloscyllium ventriosum
Scyliorhinus canicula
Scyliorhinus retifer
Apristurus melanoasper
Pseudotriakis microdon
Proscyllium habereri
Mustelus asterias
Leptocharias smithii
Hemipristis elongata
Carcharhinus acronotus
Sphyrna lewini
Eusphyra blochii
50
67
79
77
83
59
70
56
90
77
83
90
66
60
79
75
87
84
84
86
92
52
94
55
58
93
0.3 substitutions per site

Supp. Fig. 5.
